# Estimated travel time and staffing constraints to accessing the Ethiopian health care system: A two-step floating catchment area analysis

**DOI:** 10.7189/jogh.13.04008

**Published:** 2023-01-27

**Authors:** Nathaniel Hendrix, Samson Warkaye, Latera Tesfaye, Mesfin Agachew Woldekidan, Asrat Arja, Ryoko Sato, Solomon Tessema Memirie, Alemnesh H Mirkuzie, Fentabil Getnet, Stéphane Verguet

**Affiliations:** 1 Department of Global Health and Population, Harvard T.H. Chan School of Public Health, Boston, Massachusetts, USA; 2 National Data Management Center for Health, Ethiopian Public Health Institute, Addis Ababa, Ethiopia; 3 Addis Center for Ethics and Priority Setting, College of Health Sciences, Addis Ababa University, Addis Ababa, Ethiopia

## Abstract

**Background:**

Despite large investments in the public health care system, disparities in health outcomes persist between lower- and upper-income individuals, as well as rural vs urban dwellers in Ethiopia. Evidence from Ethiopia and other low- and middle-income countries suggests that challenges in health care access may contribute to poverty in these settings.

**Methods:**

We employed a two-step floating catchment area to estimate variations in spatial access to health care and in staffing levels at health care facilities. We estimated the average travel time from the population centers of administrative areas and adjusted them with provider-to-population ratios. To test hypotheses about the role of travel time vs staffing, we applied Spearman’s rank tests to these two variables against the access score to assess the significance of observed variations.

**Results:**

Among Ethiopia’s 11 first-level administrative units, Addis Ababa, Dire Dawa, and Harari had the best access scores. Regions with the lowest access scores were generally poorer and more rural/pastoral. Approximately 18% of the country did not have access to a public health care facility within a two-hour walk. Our results suggest that spatial access and staffing issues both contribute to access challenges.

**Conclusion:**

Investments both in new health facilities and staffing in existing facilities will be necessary to improve health care access within Ethiopia. Because rural and low-income areas are more likely to have poor access, future strategies for expanding and strengthening the health care system should strongly emphasize equity and the role of improved access in reducing poverty.

Ethiopians have benefited from increased investments in government-provided health care over the past two decades. One notable program was the introduction and revision of essential health services since 2005 [1]. While previous health system investments focused on higher acuity facilities in urban areas, the government more than quadrupled the number of rural health posts over the following eight years and vastly increased the number of frontline health workers [2,3]. This is one of several reasons for the increase in life expectancy and the substantial decrease in under-five mortality and the overall burden of disease in the country [4].

Despite these investments and achievements, geographic disparities and socioeconomic inequalities in mortality and health status persist. For example, immunization rates in many of the country’s poorer regions have improved slowly compared to those in wealthier regions [3,5], possibly due to less guideline-adherent care for urgent illnesses and difficulties in accessing health care [1,6-8]. Challenges with access to health care services in low- and middle-income countries (LMICs) are common; an estimated 8.6 million deaths occurred in 2016 due to difficulties accessing quality health care [9]. The burden of poor access falls disproportionately on low-income individuals and on rural residents in LMICs, consequently extending poverty cycles [10].

It is unclear how issues related to quality medical care access within Ethiopia should be addressed, or if they are the result of factors such as the location of health care facilities or their staffing levels. To guide the allocation of future health sector resources (including primary care resources), we analyzed the country-wide access to entry-level public health care. We leverage data on the distribution of health care facilities, staffing at health care facilities, the distribution of population, road infrastructure, and terrain to test our hypothesis that both physical access and staffing shortages substantially impact overall health care access in Ethiopia. The method we used to compare these factors has been validated in high-income countries to provide less biased insights on drivers of poor access compared to other available methods [11]. This work will provide information that can be used for the allocation of health care resources within Ethiopia.

## METHODS

### Overview

Using observational methods, we constructed spatial access scores that reflect both travel impedance and health care provider-to-population ratios. We first calculated the travel time from neighborhood-level population centers to nearby health facilities. Next, we computed the provider-to-population ratio by summing the number of individuals within the catchment area of each health facility and divided by the estimated number of clinicians at that specific health facility. Finally, we calculated access score by weighting health facilities by travel time using a gravity-based formula that also included the provider-to-population ratios.

### Administrative boundaries

Most decisions on health sector resource allocation in Ethiopia are made at the regional or zonal/sub-city level. We considered 11 first-level administrative units, comprising nine regions and two chartered cities. We wanted to provide information on access with the highest practical level of resolution and chose to conduct our analysis at the kebele level for all regions except the Somali region. Kebeles are the smallest administrative units in Ethiopia and are analogous to census blocks. The Somali region, however, primarily uses woredas, which are one administrative level higher than kebeles, and we therefore used woreda as the level of analysis for this region. Thus, we analyzed data for 85 woredas from the Somali region and 15 670 kebeles from the other regions. Due to data availability, we used data from the Ethiopian Water Land Resource Center on administrative boundaries that reflected those of the Southern Nations, Nationalities, and Peoples’ Region prior to the secession of Sidama and the South West Ethiopia Peoples’ Region (recently in 2019 and 2021).

### Population

We used data from WorldPop for population estimates [12] – specifically, the 2020 population estimates at the one kilometre by one kilometre resolution, adjusted so that national population equalled the United Nations projected population. WorldPop estimates the spatial distribution of population by using algorithms to detect settlements from satellite imagery. We used their top-down, unconstrained estimates, meaning that the algorithm allocates government-estimated regional population across space based on the regions’ features in satellite images [13-15].

Access to health care was calculated from the population center (rather than geographic center) of each administrative unit, as it reflects the point from which its residents are most likely to be traveling from, while the geographic center may (not) be populated. We first found the centroid of each population center from one kilometre by one kilometre square of the WorldPop data. Then, we found the population center for each administrative center by calculating the population-weighted mean at each location.

### Health facilities and providers

There are three broad categories of public health facilities in Ethiopia: health posts (generally found in rural areas and staffed by health extension workers), health centers (staffed by mid-level health care workers), and hospitals (primary, general, and referral – which have a large number of diverse staff with varying specialties) [16]. We used the Ethiopian Ministry of Health’s data on the location and type of each health facility in the country (up to date as of December 2021).

Staffing data at the health facility level was not available. However, we had access through the Ethiopian Public Health Institute to data on the number of staff at each facility type in each region. We considered the following types of health care staff to be providers for our provider-to-population ratios: health extension workers, health officers, midwives, nurses, pharmacists, general practitioners, and specialist physicians. We assigned the average number of providers at all facilities of its type within its region to each health facility (**Table 1**). Due to data limitations, we were not able to account for differences in provider capacity within or across professions.

**Table 1:** Mean staff count by region and facility type, as provided by the Ethiopian Public Health Institute

Region†	Health post	Health center	Hospital
Addis Ababa	NA	68.3	308.2
Afar	1.8	6.8	58.4
Amhara	2.5	21.5	164.1
Benishangul Gumuz	2.3	19.2	135.2
Dire Dawa	5.6	34.8	196.2
Gambela	4.7	22.2	84.2
Harari	4.6	29.4	102.0
Oromia	2.3	14.0	139.8
Southern Nations, Nationalities, and Peoples’ Region	2.3	18.4	118.4
Somali	2.2	13.4	167.3
Tigray	2.7	27.1	100.1

NA – not applicable

*Staff counts were used to calculate provider-to-population ratios.

†Administrative regions prior 2019, before the creations of the Sidama region (2019) and of the South West Ethiopia Peoples’ Region (2021).

### Travel times

We calculated travel time between population centers and nearby health facilities using a four-step algorithm. We first identified all health facilities within a 10-km radius of the population center. Next, a friction surface map was created based on the Malaria Atlas Project in 2020 to account for barriers to travel such as rivers, vegetation covers, and elevated areas, as well as travel facilitators such as roads [17]. The friction surface map indicates the reduction from the assumed optimal walking speed of five kilometres per hour caused by various challenges, such as change in elevation and surface type. We used this friction surface map to calculate travel times over longer distances in the analysis.

Some travel times could not be calculated with the friction surface map when the population center and health facility were in the same data cell of the friction surface map. In such cases, we used the Google Maps application programming interface (API) to calculate travel times [18]. However, this API was limited as it could only calculate travel time along roads and not travel times for some routes, while it occasionally produced unexpectedly long travel times due to highly indirect routes. In these few cases, we estimated travel time using Euclidean distance – that is, a straight line between two points – multiplied by an assumed walking speed of 2.5 km/h.

### Spatial access scores

We used a two-stage, gravity-based model to score spatial access to health care, largely following the methods of Cao et al. [19]. The first step was defining the provider-to-population ratio. We defined the catchment area as a circle around the health facility with a radius of 10 km. We estimated the population within the catchment area by weighting the population at each nearby population center with a Gaussian function of travel time (*t*) and a gravity decay coefficient (β) – i.e. using the formula *w = e^-t2/β^*. We assigned β such that a two-hour travel time had a weight of 0.01.

For each health facility (*h*) with *s* providers employed nearby population centers with population *p_i ∈ {1,…,n}_,* we used the following formula for provider-to-population ratio (*r*):



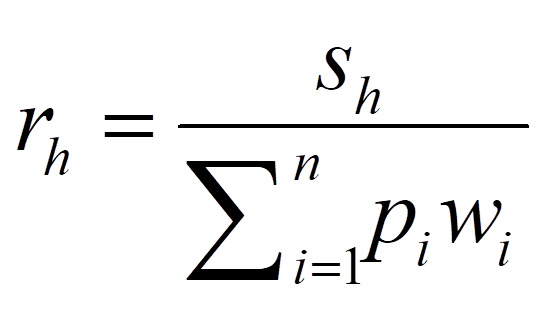



We then calculated the access score (*a*) for each population center with nearby health facilities *h_j ∈ {1,…,k}_* using the following formula, which includes travel time (*w*) and the provider-to-population ratio (*r*) calculated above:



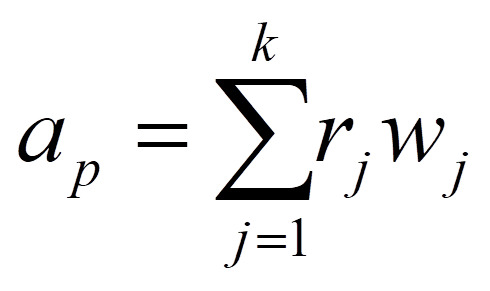



For ease of representation and to account for uncertainty stemming from the resolution of the friction surface map, we first divided population centers into those with a health facility within a two-hour walk and those without (denoted “no access” for simplicity). We further divided the population centers with access to a health facility by access score into quintiles because the scores have only a relative interpretation, rather than any interpretable absolute meaning.

To summarize inequalities in accessibility, we tentatively calculated the population weighted average of administrative area scores for each region. Because the Somali region did not have kebele-level data, we also conducted a woreda-level analysis to better contextualize health care access within the Somali region.

Finally, we tried to identify whether travel impedance or human resource constraints were primarily responsible for the observed differences in scores by using a Spearman rank test to assess the significance of the association between access score and administrative unit population and the association between access score and travel time to the nearest health facility.

### Ethics statement

Ethical approval was not required for this study because it did not use any patient data. We received permission from the Ethiopian Water Land Resource Center and Ethiopian Public Health Institute to use their data. Malaria Atlas Project data are freely available for public use and do not require permission to use.

## RESULTS

Our analysis covered 15 722 administrative units with a total population of approximately 104 million. Kebeles had an average population of 6201 (standard deviation (SD) = 9338), and woredas had an average population of 139 981 (SD = 89 163). Among these administrative units, 2968 had no access to a health facility within a two-hour walk of their population centers (**Table 2**). Administrative units with health care access had a mean access score of 0.0044 and median access score of 0.0044. Access scores ranged from 0.0001 to 5.2680, although these scores should only be interpreted in relation to other scores within this study.

**Table 2:** Distribution of population and access scores across administrative units by access category*

Access category	Number of administrative units	Total population	Mean population per unit	Standard deviation of population	Mean access score per unit (range)	Standard deviation of access score (IQR)
Best access	2551	11 909 600	4700	5500	0.0481 (0.0099-5.2680)	0.2070 (0.0124-0.0325)
Good access	2551	15 317 300	6000	8900	0.0072 (0.0054-0.0099)	0.0013 (0.0061-0.0082)
Moderate access	2550	18 462 300	7200	11 100	0.0044 (0.0035-0.0054)	0.0006 (0.0039-0.0049)
Poor access	2551	18 671 600	7300	14 400	0.0027 (0.0018-0.0035)	0.0005 (0.0022-0.0031)
Very poor access	2551	20 361 100	8000	17 900	0.0010 (0.0001-0.0018)	0.0005 (0.0005-0.0014)
No access	2968	19 125 900	6400	14 000	NA	NA

IQR – interquartile range, NA – not applicable

*Administrative units were first divided by whether they had access to any health facility within a two-hour walk (“access”); those were then divided into quintiles by access score.

**Figure 1:**
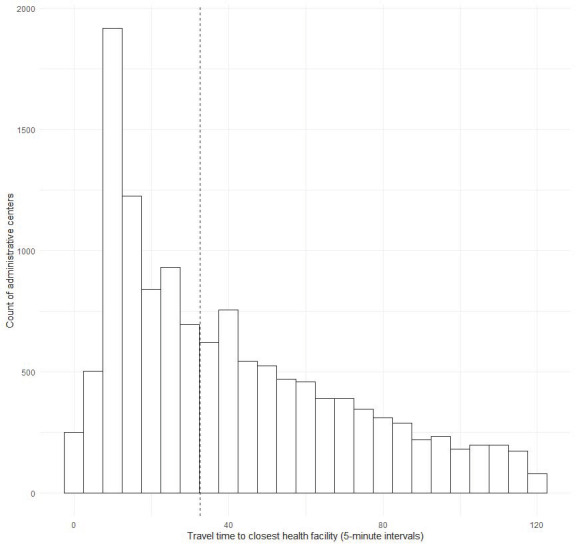
Distribution of walking time from population centers (n = 12 754) to the most accessible health facility. The vertical dashed line represents the median time of 33 minutes. Population centers with walking time to the most accessible health facility in excess of two hours are not shown.

The average walking time from the population center to the most accessible health facility for administrative units with access to health care was 41 minutes and the median time was 33 minutes (**Figure 1**). The distribution of travel times was right skewed in regions with higher access scores, but relatively uniform in regions with poorer access (Figure A1 in the **Online Supplementary Document**).

**Figure 2:**
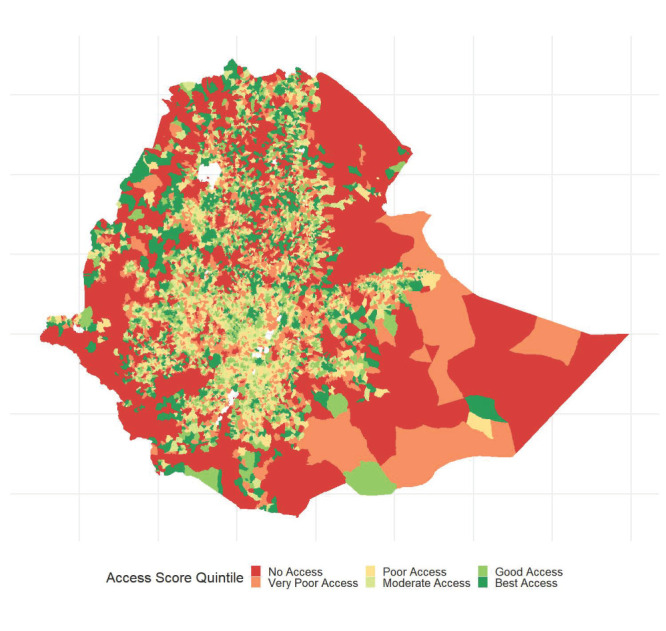
Map of access to health care in Ethiopia. Administrative units are kebeles (fifth level administrative units or neighborhoods) for all regions except Somali, which uses woredas (fourth level administrative units or districts). Uncolored areas of the map within the borders of Ethiopia are bodies of water. No access corresponds to no access to a health facility within a two-hour walk of population centers.

We found clear regional differences in health care access (**Figure 2**, **Table 3**). Addis Ababa, Dire Dawa, and Harari have the highest access scores. These are all small regions that center on relatively dense urban areas. The regions of Afar, Somali, and the Southern Nations, Nationalities, and Peoples’ (SNNP) region have relatively low access.

**Table 3:** Population-weighted average access scores for administrative units within each region*

Region†	Kebele-level score	Woreda-level score
Addis Ababa	154	2266
Afar	21	177
Amhara	57	1160
Benishangul Gumuz	56	454
Dire Dawa	114	2787
Gambella	49	102
Harari	145	3072
Oromia	29	947
Southern Nations, Nationalities, and Peoples’ Region	28	1283
Somali	NA	115
Tigray	84	1100

NA – not applicable

*Access scores are purely relative and have no absolute interpretation. Kebeles are fifth-level administrative units and woredas are fourth-level administrative units.

†Administrative regions prior to 2019, before the creations of the Sidama region (2019) and of the South West Ethiopia Peoples’ Region (2021).

The average population of administrative units with access scores in the top quintile was 4700 (SD = 5500), while administrative units in the bottom quintile of access had a mean population of 8000 (SD = 17 900). The population of administrative units was inversely associated with access score (*P* < 0.001). Similarly, we observed an inverse correlation between access score and travel time to the closest health facility (*P* < 0.001).

## DISCUSSION

This study is one of the first to apply contemporary access algorithms to a low- or middle-income country. Our results suggest that relatively extreme disparities in access exist. Approximately 18% of Ethiopia’s population does not have access to a health care facility within a two-hour walking distance. We observed significant inverse correlations between population and access score, and minimum travel time and access score. This suggests that travel impedance and staffing both contribute to the observed variations in access scores.

Besides the public health facilities we included in this analysis, Ethiopia has a robust system of private health care clinics concentrated in urban settings, numbering approximately 4000 compared to 18 000 public facilities [20]. Our focus on the latter was due to their affordability, since cost is a major barrier to access [21]. However, proximity, waiting times, or perceived quality may cause individuals to prefer private clinics [22]. Consequently, the costs of seeking care at private clinics may increase the financial burden of diseases. The financial risk associated with poor access to public facilities is an understudied issue [23].

Difficulties with accessing health care are an important contextual determinant of health, which is often associated with decisions made outside of the health care system, such as decisions about where to build roads or the resources allocated to deploy clinicians or construct and equip health facilities [24]. Resolving access barriers will thus require a holistic vision of health that encompasses sectors other than health care.

Access limits efforts to improve equity and reduce the financial burden of diseases; it also results in resource misallocation, when plans are made according to cost-effectiveness analysis only [25]. This is because cost-effectiveness analyses usually assume a certain level of accessibility to the interventions that they focus on. When a health care service/facility is difficult to access for the population of a given area, the results of a cost-effectiveness analysis may not generalize to these areas [23]. Another issue is that human resources barriers may limit the cost-effectiveness of interventions due to limits on clinicians’ capacity to add to their current responsibilities [26,27]. Combined, these factors may hinder the feasibility of effectively carrying out certain interventions.

Many LMICs face challenges while providing equitable access to health care services. Spatial access has been the primary access challenge in studies examining subjects such as cervical cancer screening in Nigeria [28], contraceptive use in the Democratic Republic of Congo [29], and emergency care in Bangladesh [30]. However, human resources were shown to be the primary constraint in surgical access in Ghana and non-emergency care in Zambia [31,32]. This variation in the cause of access challenges shows the necessity of location-specific and (potentially) service-specific analyses for determining how best to improve health care access in a targeted manner.

The methods we have used in this research have primarily been applied in high-income countries due to the requirement of highly detailed data. For example, it was used to better understand barriers to access to opioid use disorder treatment in the state of New Hampshire in the USA [19]. Similar methods were also used to allocate automated external defibrillators in the Taiwanese city of Kaohsiung [33]. There are, however, notable differences in the approach used in these contexts. As the research from Taiwan shows, highly detailed data often allow for more granularity in characterizing the places of interest: the researchers were able to measure the percent of elderly people in each area and to account for different types of land use. In the study on opioid use disorder treatment in New Hampshire, the analyses also consider different types of transportation use. While we used walking speed in our study, analyses in high-income countries generally assume that people will drive or take public transit to health services instead.

This study has several limitations. First, data on staffing at individual health facilities was not accessible. To work around this limitation, we had to use region-level data on health care staff averaged by facility type. This may have resulted in overestimating population staffing at some facilities and underestimating it at others. That said, our estimates are generally aligned to those of other studies [34]. We were also limited by the resolution of the friction surface map, which prevented us from precisely calculating travel times over short distances; we thus resorted to Google Maps and estimates of travel time using Euclidean distances for some health facilities that are close to population centers. These less precise methods may have limited our ability to distinguish between the access scores of population centers with nearby health facilities. This potential source of error is one reason we primarily discussed access scores in terms of quintiles. A final limitation came from the population data: WorldPop allows for more granular, detailed estimates of the population distribution than government statistics do [12]. However, these are based on satellite images that estimate the degree of human settlement and the associated density. This method is known to slightly overestimate the population of sparsely populated regions while underestimating the population of dense areas [13]. As such, this data source may have led us to misallocating some population.

## CONCLUSIONS

We identified patterns in health care access within rural areas of Ethiopia that would require investments both in new health facilities located in areas with poor or no access and increased staffing at existing health facilities. The best access within the country is in relatively wealthy and dense urban areas, while more rural and low-income areas are more likely to have no access to health care [21]. As such, the role of health care access in potentially extending poverty cycles should be considered in future health care priority setting and intersectoral poverty reduction programs.

## Additional material


Online Supplementary Document
