# Supplementary material for: Estimated travel time and staffing constraints to accessing the Ethiopian health care system: A two-step floating catchment area analysis
Source: J Glob Health. 2023 Jan 27;13:04008. doi: 10.7189/jogh.13.04008 (PMC9880518; doi:10.7189/jogh.13.04008)
Supplement: Online Supplementary Document [file jogh-13-04008-s001.pdf]

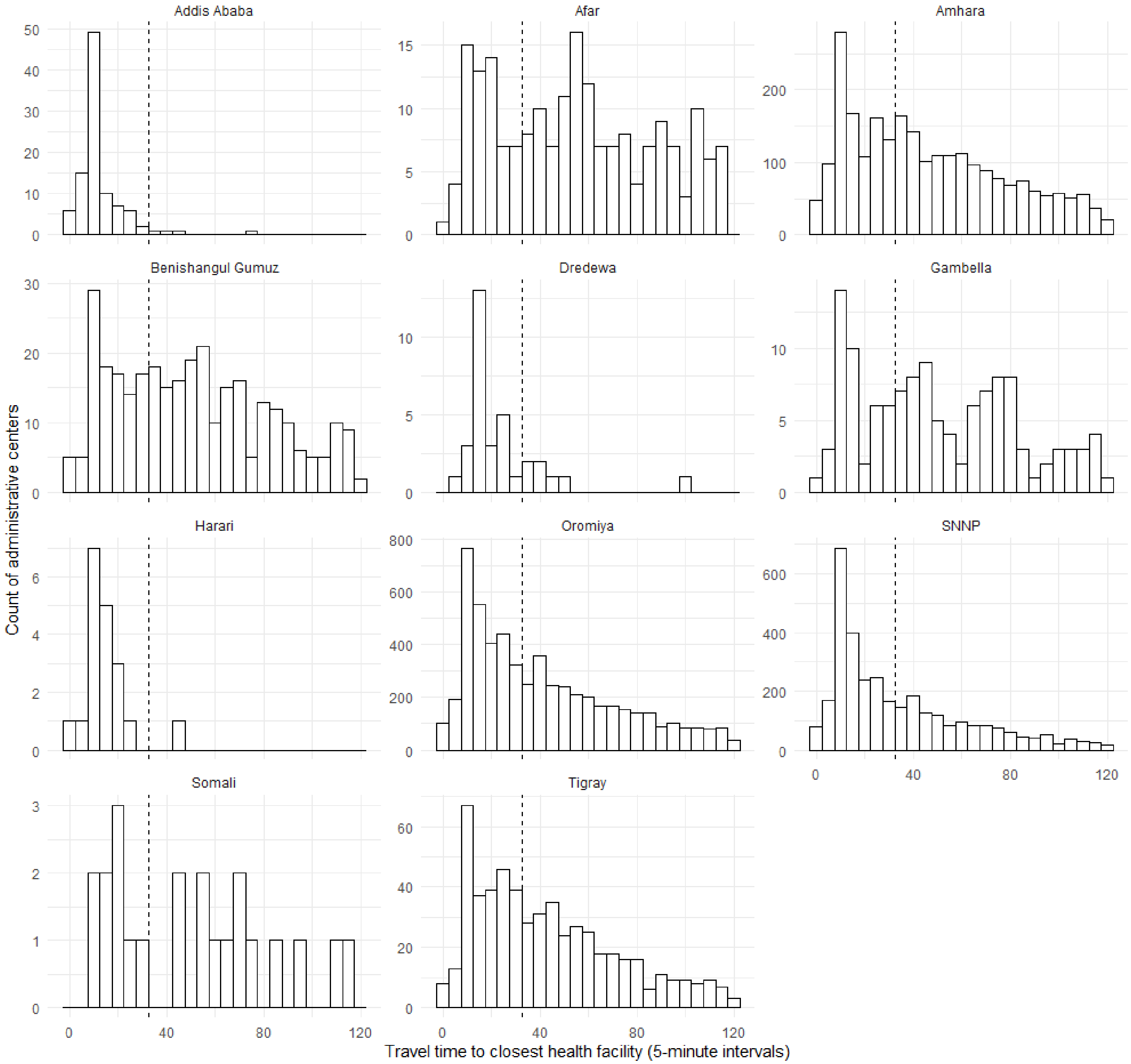

Appendix Figure A1: Distribution within regions of walking time from population centers to the nearest health facility. Population centers are defined by woreda (district) for the Somali region and by kebele (neighborhood) for all other regions. The dotted line represents the national median travel time from population centers to the nearest health facilities of 33 minutes.

SNNP = Southern Nations, Nationalities, and Peoples' Region

Note: Administrative regions prior 2019, before the creations of the Sidama region (2019) and of the South West Ethiopia Peoples' Region (2021).
